# Pericardial fat and its influence on cardiac diastolic function

**DOI:** 10.1186/s12933-020-01097-2

**Published:** 2020-08-17

**Authors:** Vera H. W. de Wit-Verheggen, Sibel Altintas, Romy J. M. Spee, Casper Mihl, Sander M. J. van Kuijk, Joachim E. Wildberger, Vera B. Schrauwen-Hinderling, Bas L. J. H. Kietselaer, Tineke van de Weijer

**Affiliations:** 1 NUTRIM School of Nutrition and Translational Research in Metabolism, Maastricht University, Maastricht, Netherlands; 2 Department of Nutrition and Movement Sciences, Maastricht University Medical Center, Maastricht, Netherlands; 3 Department of Cardiology, Maastricht University Medical Center, Maastricht, Netherlands; 4 Department of Radiology and Nuclear Medicine, Maastricht University Medical Center, Maastricht, Netherlands; 5 CARIM School for Cardiovascular Diseases, Maastricht University Medical Center, Maastricht, Netherlands; 6 Department of Clinical Epidemiology and Medical Technology Assessment, Maastricht University Medical Center, Maastricht, Netherlands

**Keywords:** Pericardial fat, Epicardial fat, Cardiac diastolic function

## Abstract

**Background:**

Pericardial fat (PF) has been suggested to directly act on cardiomyocytes, leading to diastolic dysfunction. The aim of this study was to investigate whether a higher PF volume is associated with a lower diastolic function in healthy subjects.

**Methods:**

254 adults (40–70 years, BMI 18–35 kg/m^2^, normal left ventricular ejection fraction), with (a)typical chest pain (otherwise healthy) from the cardiology outpatient clinic were retrospectively included in this study. All patients underwent a coronary computed tomographic angiography for the measurement of pericardial fat volume, as well as a transthoracic echocardiography for the assessment of diastolic function parameters. To assess the independent association of PF and diastolic function parameters, multivariable linear regression analysis was performed. To maximize differences in PF volume, the group was divided in low (lowest quartile of both sexes) and high (highest quartile of both sexes) PF volume. Multivariable binary logistic analysis was used to study the associations within the groups between PF and diastolic function, adjusted for age, BMI, and sex.

**Results:**

Significant associations for all four diastolic parameters with the PF volume were found after adjusting for BMI, age, and sex. In addition, subjects with high pericardial fat had a reduced left atrial volume index (p = 0.02), lower E/e (p < 0.01) and E/A (p = 0.01), reduced e′ lateral (p < 0.01), reduced e′ septal p = 0.03), compared to subjects with low pericardial fat.

**Conclusion:**

These findings confirm that pericardial fat volume, even in healthy subjects with normal cardiac function, is associated with diastolic function. Our results suggest that the mechanical effects of PF may limit the distensibility of the heart and thereby directly contribute to diastolic dysfunction.

*Trial registration* NCT01671930

## Background

Diastolic heart failure is a major cause of morbidity and mortality [1] and is preceded by diastolic dysfunction, which is often present in patients with obesity and type 2 diabetes mellitus (T2DM). Diastolic dysfunction is defined as abnormal relaxation of the myocardium and may be present years before symptoms occur. It can be diagnosed by quantifying diastolic tissue motion and intracavitary filling pressures. The guidelines for diagnosing diastolic function combine measurement of diastolic tissue motion, diastolic blood flow quantification, and structural abnormalities such as the presence of left atrial dilation [2]. Meeting 2 or more criteria results in the diagnosis of diastolic dysfunction.

Despite the clear definition, the understanding of the pathological mechanism of diastolic dysfunction remains poor. Various potential mechanisms have been suggested, but none of them can adequately explain the pathological process. Since increased pericardial fat (PF) volume is associated with adverse cardiovascular disease (CVD) outcomes, interest has peaked into this relationship and the potential effects of PF on cardiac dysfunction [3, 4].

PF is divided into two fat components: the Epicardial Adipose Tissue (EAT) and the Cardiac Adipose tissue (CAT). It is presumed that the EAT, due to its anatomical proximity to the myocardium, has the most effects on the myocardium. In normal physiology, EAT may have positive metabolic effects as it has an important function in lipid storing, and it also secretes endocrine factors [5]. It demonstrates a great flexibility in the storage and release of fatty acids, which has been suggested to protect the heart from lipotoxicity, whilstsimultaneously providing energy to the myocardium during high energy demand [6, 7]. As a metabolically active endocrine organ, EAT also produces adipokines which may protect the heart from cardiovascular disease [8]. However, when EAT expands, the balance between the storage and release of fatty acids shifts towards a more active secretion, as seen in obese subjects in comparison to lean subjects [9]. The expanded EAT transforms its secretions into pro-inflammatory cytokines and chemokines [6, 8, 10]. Cho et al. showed that the thickness of EAT at the right ventricle wall was associated with inflammation represented by hs-CRP level, LV mass, and subclinicial myocardial dysfunction in males [11]. This is also confirmed in EAT biopsies taken from patients undergoing coronary artery bypass grafting (CABG) [12, 13]. Some of these mediators are known to have profibrotic properties, linking the inflammation of enlarged EAT with fibrosis [14]. From studies performed in (morbidly) obese subjects with a high prevalence of T2DM, we know that PF, EAT, and CAT are linked to several diastolic function parameters [15–17]. However, studies associating PF directly with diastolic function in healthy subjects are scarce, and the underlying mechanisms remain unknown [18–21].

Moreover, Ng et al. found an association between EAT volume index and interstitial myocardial fibrosis in an overweight to obese population [19]. This association suggests that enlarged EAT may be related to asymptomatic cardiac remodeling, and hence, the enlarged EAT may be involved in the development of cardiac diastolic dysfunction as is seen in overweighed subjects. Most studies on EAT have focused on the effects of EAT on systolic function, whereas in fact, in obese and diabetic populations, diastolic function are the first cardiac function parameters to change in obesity and metabolic syndrome [22]. In addition, Yang et al. showed an increased EAT burden in pre-diabetic and diabetic subjects, compared to normoglycemic subjects [23]. Also, Christensen et al. found that high levels of EAT were associated with the composite of incident CVD and mortality in subjects with T2DM [24]. EAT may possibly play a more central role in the development of asymptomatic diastolic cardiac dysfunction than previously assumed, underlining the importance of a better understanding of the relationship between EAT and early changes in cardiac diastolic function. Hence, further studies focusing on exploring the relationship between EAT and diastolic dysfunction in a relatively healthy population, independently of their metabolic profile, are warranted.

In summary, it is unknown whether PF and/or EAT influences diastolic cardiac function in healthy subjects before any symptoms of diastolic failure occur. Most studies looking into the associations between PF or EAT with diastolic function have been performed in subjects with heart failure, CVD, overweight, or (pre-)diabetes [18–20, 25]. This may possibly confound the relationship, as many structural and metabolic changes may interfere. Therefore, in this study, we aim to determine whether a higher PF volume is associated with subclinical but lower diastolic function in a healthy population. Secondly, we aim to examine if this lower diastolic function is solely derived from the EAT compartment, or if it is associated to the PF compartment as a whole.

## Methods

### Study cohort

This study was approved by the Institutional Review Board (IRB) and Ethics Committee. Involved data were collected on a routine basis from within the Maastricht biomarker CT study (ClinicalTrials.gov NCT01671930, MEC 08-4-057) and analysed anonymously in accordance with Institutional Review Board guidelines. The study complies with the ethical principles of the Helsinki Declaration.

This study cohort is comprised of patients from the cardiology outpatient clinic presenting with (a)typical chest pain, who were according to the standard care protocol referred for coronary computed tomographic angiography (CCTA) for the evaluation of stable CVD, in accordance with the current guidelines [26, 27]. Inclusion criteria for the Maastricht biomarker CT study were a recent history of cardiac typical or atypical chest pain, dyspnea, or collapse; at least 1 mL of serum for determination of biomarkers; and a diagnostic CCTA-scan, defined as 7 or more interpretable coronary segments. The exclusion criteria were hsCRP concentration ≥ 10 mg/L (indicating underlying inflammatory disease), severe renal dysfunction, or dialysis (due to application of contrast fluids).

**Fig. 1:**
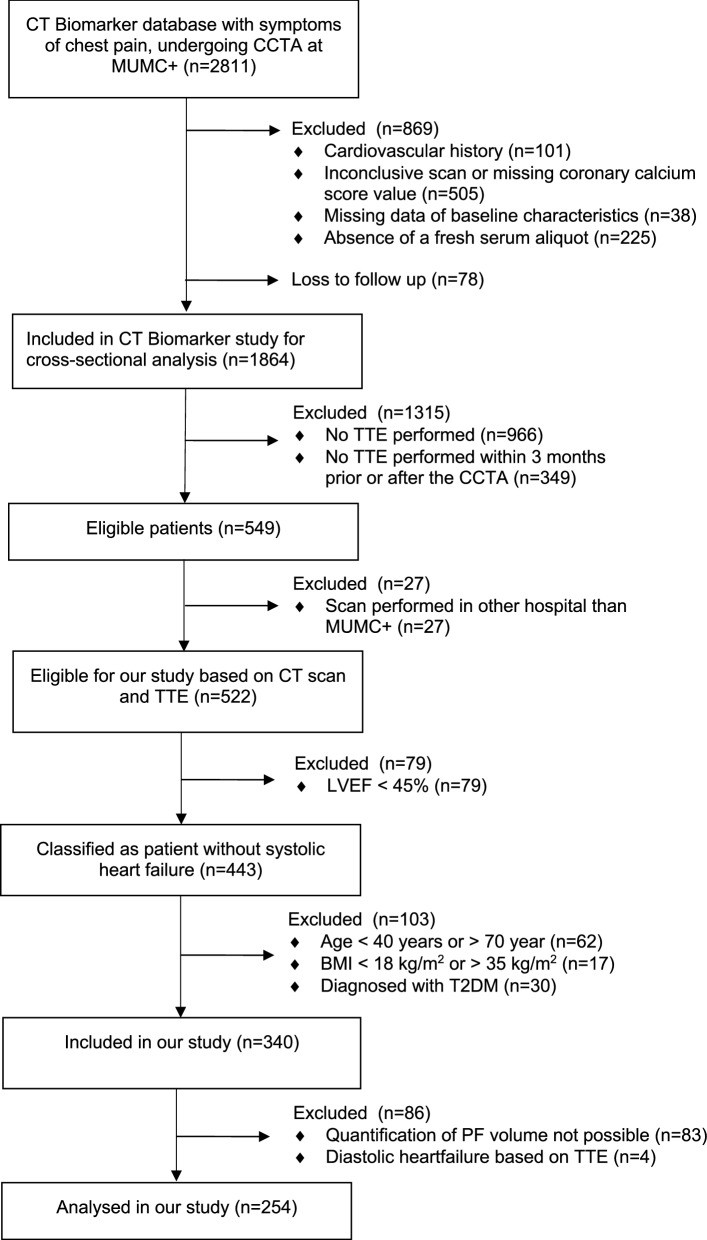
Flowchart of inclusion (n = 254). 254 patients from the Maastricht biomarker CT study were eligible for the analysis of the association of PF and diastolic function in healthy subjects

254 patients enrolled in the echocardiography subgroup of the Maastricht Biomarker CT study were retrospectively included in this study [28, 29]. A flowchart of inclusion is provided in Fig. 1. In the present subgroup analysis (n = 254), patients aged 40-70 years with a BMI between 18 and 35 kg/m^2^ without history or diagnosis of acute coronary syndrome at the time of CCTA were included. Exclusion criteria for this subgroup study were: left ventricular ejection fraction (LVEF) < 45%, diastolic dysfunction, atrial fibrillation, and diabetes mellitus.

### Biochemical analysis

Serum samples were collected just before CCTA, processed within 2 h and directly stored at − 80 °C until analysis. Total cholesterol (CV 2.0%), triglycerides (CV 2.5%), high-density (CV 3.0%) and low-density lipoprotein concentrations were measured as previously described (Cobas 6000, Roche Diagnostics) [28]. Serum creatinine (CV 2.5%) and cystatin C concentrations were measured in a fresh aliquot (Cobas 6000; Roche Diagnostics). Creatinine concentrations were assessed using the enzymatic method (Cobas 6000, Roche Diagnostics). Cystatin C was measured using a new particle-enhanced turbidimetric assay (Gentian AS), which was standardized against the certified ERM-DA471/IFCC cystatin C reference material [30]. Glomerular filtration rate was estimated by the Chronic Kidney Disease Epidemiology Collaboration equations using serum creatinine and cystatin C concentrations [31].

### Cardiac computed tomographic angiography

All 254 patients had undergone a standardized non-enhanced scan to determine the calcium score using the Agatston method [32] at our center prior to CCTA assessment.

**Fig. 2:**
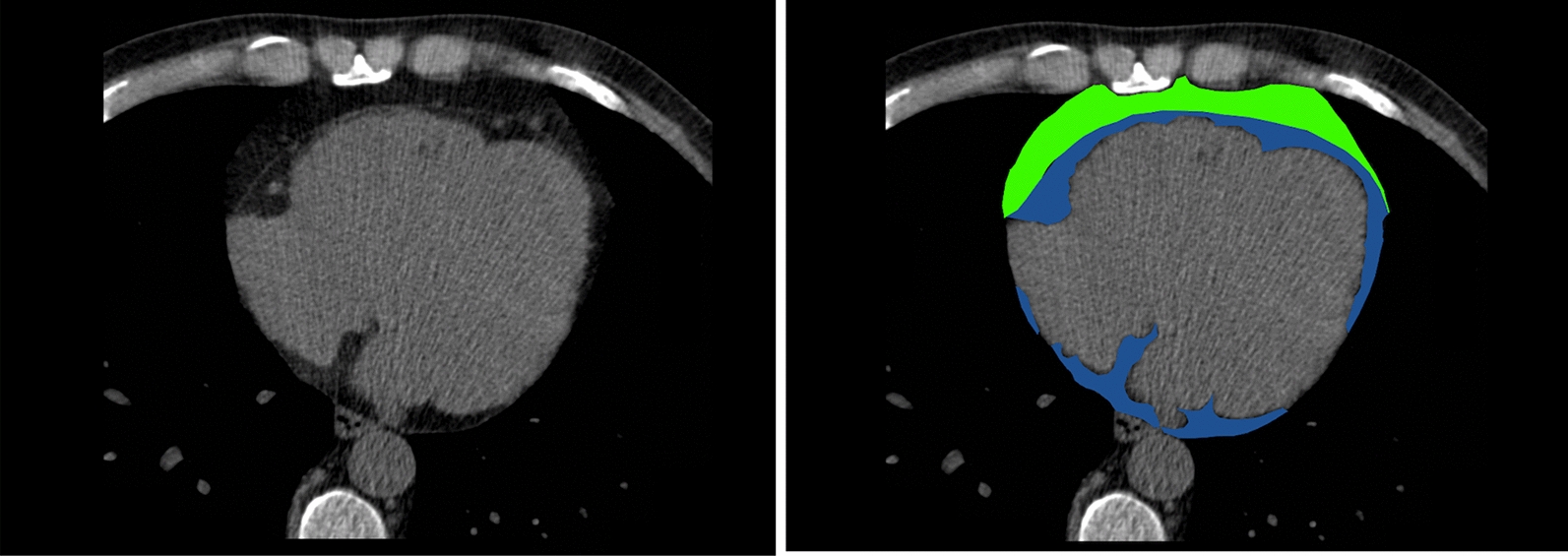
Definition of pericardial fat (PF) and the related adipose tissues. The adipose tissue surrounding the heart is defined as the pericardial fat (PF) and is a combination of epicardial and cardiac fat components. Within the PF, the pericardium demarcates the epicardial adipose tissue (EAT) from the cardiac adipose tissue (CAT). EAT (depicted in blue) is located between the myocardium and visceral pericardium, CAT (depicted in green) is located adherent and external to the parietal pericardium

Semi-automatic segmentation determined the PF volume by dedicated software (SyngoVia, Siemens Healthineers, Forcheim, Germany) using a threshold from − 150 to − 50 Hounsfield Units to distinguish visceral adipose tissue, as set by the software [33]. Because of the large sample size, only in a random sample of 10% of the subjects the pericardium was marked manually to separate the PF into EAT and CAT (depicted in Fig. 2), and thereafter, the software calculated the separate 3D volumes of EAT and CAT.

### Echocardiography

Echocardiography was performed within a period of 3 months from the CCTA by an experienced echocardiographist. Transthoracic images of the left ventricle (LV) were acquired to assess morphology, function and mass (Philips IE 33, Philips Healthcare). LV function and -mass were calculated by off-line analysis using Xcelera software package (Philips), according to current ESC/AHA guidelines [34].

Only four diastolic parameters are decisive in the evaluation of diastolic function according to the American Society of Echocardiography (ASE)/European Association of Cardiovascular Imaging (EACVI) guidelines, namely, left atrial volume index (LAVI), e′ septal, e′ lateral (mobility of the septal and lateral left ventricle wall respectively), and peak velocity of tricuspid regurgitation (TR) [2]. Therefore, most of the analyses will focus upon these diastolic function parameters. But, in addition, also mitral peak A and E velocity, E/A ratio, and E/e′ ratio, were determined.

### Statistical analysis

Baseline characteristics of the sample were summarized using mean and standard deviation or median and interquartile range (IQR) for normally distributed and skewed continuous variables, respectively. Categorical data were presented as absolute number and percentage. To assess the independent association of PF and diastolic function parameters in these 254 patients, linear regression analysis was performed with either LAVI or e′ septal or e′ lateral or TR as the dependent variable. These models were adjusted for BMI, age, sex, and their interaction terms with PF, since it is known that these parameters are strongly associated with PF [9, 35, 36]. Results of the linear regression analysis are presented as regression coefficient with 95% confidence interval (95% CI).

This study is based on a sample of healthy participants without diastolic dysfunction, therefore, only mild differences in diastolic function were expected. To maximize the differences in PF volume, the group was divided into low PF (lowest quartile of both sexes separately) and high PF (highest quartile of both sexes separately). The lowest and highest quartile groups were matched for cardiovascular risk factors, i.e., sex, systolic and diastolic blood pressure, total and LDL cholesterol, and kidney function. Differences in other baseline characteristics across these extreme quartiles of PF volume were investigated using the independent-samples *t* test for continuous variables with a normal distribution, or the Mann–Whitney U-test for non-normal distributed continuous variables. Pearson’s Chi square test was used for categorical variables. Data are presented as proportions, mean ± standard deviations, and data with a non-normal distribution are presented as the median (interquartile range, IQR).

To assess the independent association of PF and diastolic function parameters in these extreme quartiles (n = 130), also multivariable linear regression analysis was performed with either LAVI, or e′ septal, or e′ lateral, or E/e′, or TR as the dependent variable. These models were adjusted for BMI, age, and sex. Results are presented as regression coefficient with 95% confidence interval (95% CI).

To investigate the association of EAT with the total PF and EAT with diastolic function, Pearson’s correlation coefficient was computed. Because only in 10% of the subjects an EAT volume was known, this subgroup was considered too small to perform regression analysis. All statistical analyses were performed with IBM SPSS Statistics Version 25.0 (SPSS, Inc.). Two-sided p-values of ≤ 0.05 were considered statistically significant.

## Results

The baseline characteristics for the total sample and the lowest and highest quartile groups of PF volume are presented in Table 1.

**Table 1:** Baseline characteristics of the study sample, and divided into highest and lowest quartiles of PF

	Total sample (n = 254)	PF low (n = 65)	PF high (n = 65)	p-value
Demographics				
Age (years)	57.0 ± 7.5	55.7 ± 8.0	59.1 ± 7.4	0.015
Sex (% female)	48	46	48	0.860
Cardiovascular risk factors				
Framingham Risk Score	18.0 ± 13.2	14.4 ± 10.1	21.4 ± 16.1	0.004
Glucose (mmol/L)	5.6 ± 0.9	5.5 ± 0.8	5.9 ± 1.2	0.025
Body mass index (kg/m^2^)	26.4 ± 3.7	23.7 ± 2.7	28.1 ± 2.9	< 0.001
Systolic bloodpressure (mmHg)	142 ± 20	141 ± 23	146 ± 20	0.139
Diastolic bloodpressure (mmHg)	81 ± 11	80 ± 12	82 ± 11	0.254
Total cholesterol (mmol/L)	5.6 ± 1.1	5.5 ± 1.2	5.8 ± 1.2	0.148
HDL cholesterol (mmol/L)	1.3 ± 0.4	1.5 ± 0.4	1.2 ± 0.4	0.001
LDL cholesterol (mmol/L)	3.6 ± 1.0	3.4 ± 1.0	3.6 ± 1.1	0.405
Triglycerides (mmol/L)	1.5 (1.0, 2.2)	1.2 (0.8, 1.5)	1.7 (1.3, 2.5)	< 0.001
Creatinine (μmol/L)	76 ± 17	76 ± 15	75 ± 18	0.769
eGFR (MDRD) (mL/min/1.73 m^2^)	88 ± 18	89 ± 16	90 ± 21	0.619
CRP (mg/L)	2.3 ± 2.7	2.1 ± 2.5	2.8 ± 3.8	0.470
Coronary artery disease				
No Plaque (%)	39.4 ± 4.9	46.2 ± 5.0	35.4 ± 4.8	0.215
Mild (%)	37.0 ± 4.8	33.8 ± 4.8	36.9 ± 4.9	0.716
Moderate (%)	10.20 ± 3.0	7.7 ± 2.7	10.8 ± 3.1	0.548
Severe (%)	11.8 ± 3.2	9.2 ± 2.9	15.4 ± 3.6	0.289
Multi-vessel (%)	1.6 ± 1.3	3.1 ± 1.7	1.5 ± 1.2	0.563

Data are presented as mean ± standard deviation, percentage, or as median (interquartile range, IQR)

### Distribution and determinants of the PF volume

**Fig. 3:**
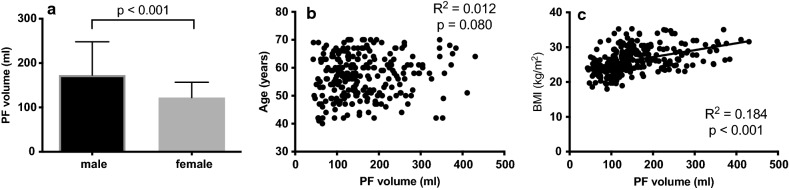
The variation of PF volume to sex, age and BMI in a healthy population. PF volume is higher in males as in females (**a**), PF volume is not related to age (**b**) and PF volume is associated with BMI (**c**)

Median (IQR) PF volume in the total cohort were 1.411 (IQR 1.035, 2.057) dl. Since males have a higher PF volume than females (median 1.729 dl, IQR 1.202, 2.492; median 1.215 dl, IQR 0.909, 1.552; respectively), the upper and lower PF volume quartiles of males and females were combined for the analysis (Fig. 3a).

There was a significant difference between the lowest and highest quartile groups for age (55.7 ± 8.0 versus 59.1 ± 7.4, p = 0.015), BMI (23.7 ± 2.7 versus 28.1 ± 2.9, p value < 0.001), glucose (5.5 ± 0.8 versus 5.9 ± 1.2, p = 0.025), HDL cholesterol (1.5 ± 0.4 versus 1.2 ± 0.4) and triglycerides (1.5 ± 1.1 versus 2.4 ± 2.0, p = 0.001), see Table 1. The CAD findings were not different between the two groups of high and low PF. However, Framingham Risk Score was higher in the high PF group, possibly due to the association of PF with age and BMI.

### Distribution and determinants of diastolic function

**Fig. 4:**
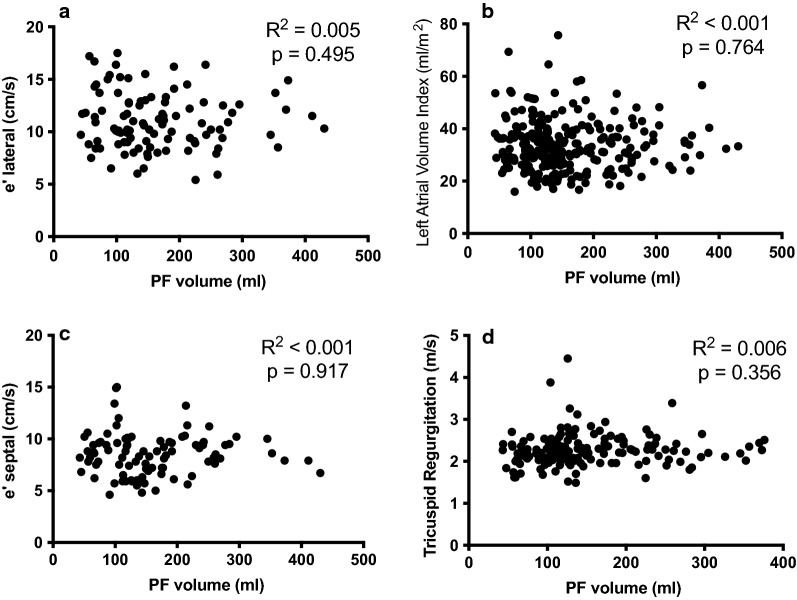
PF is not associated with diastolic function parameters in a healthy population. Data of the entire cohort (n = 254) are displayed. No correlations are found

The association between diastolic function and PF volume was investigated, as some of the diastolic parameters are expected to deteriorate during the development of diastolic dysfunction before clinical criteria for diastolic dysfunction are met (Fig. 4).

Although still in the normal range, significant differences in the diastolic function parameters were found between the lowest and highest PF quartiles. As shown in Table 2, a reduced LAVI and E/e′ was found in the lowest PF quartile (p = 0.02, p < 0.01, respectively); and a reduced e′ lateral, e′ septal, and E/A in the highest PF quartile (p < 0.01, p = 0.03, p = 0.01, respectively); and an increased peak A velocity in the highest PF quartile (p < 0.01). Peak E velocity and TR did not differ significantly between the two extreme PF volume quartiles. Together, these differences reflect a diminished, although still normal, diastolic cardiac function in the highest PF quartile compared to the lowest PF quartile.

**Table 2:** Cardiac function measured by transthoracic echocardiography

	Total population (n = 254)	PF low (n = 65)	PF high (n = 65)	p-value
Left ventricular ejection fraction (%)	61 ± 5	62 ± 5	61 ± 5	0.213
Left ventricular mass index (g/m^2^)	84.7 ± 16.9	80.6 ± 15.6	88.0 ± 16.0	0.008
Left atrial volume index (mL/m^2^)	33.7 ± 0.7	36.8 ± 10.3	32.7 ± 8.4	0.015
e′ lateral (cm/s)	11.0 ± 2.7	12.2 ± 2.9	10.3 ± 2.0	0.005
e′ septal (cm/s)	8.5 ± 2.0	9.5 ± 2.1	8.4 ± 1.8	0.034
E/A	1.1 ± 0.4	1.1 ± 0.4	1.0 ± 0.4	0.013
Peak E velocity (cm/s)	72 ± 20	73 ± 24	70 ± 18	0.425
Peak A velocity (cm/s)	72 ± 18	66 ± 16	74 ± 17	0.004
E/e′	7.9 ± 2.1	6.8 ± 1.7	8.3 ± 2.3	0.009
Tricuspid regurgitation (m/s)	2.3 ± 0.4	2.2 ± 0.4	2.3 ± 0.3	0.416

Data are presented as means ± standard deviation

Reference values: LVEF ≥ 45%, LAVI < 34 ml/m^2^, e′ lateral > 10 cm/s, e′ septal > 7 cm/s, E/A 0.8–2.5, E/e′ 8–14, TR 2.0–2.8 m/s

### Association of PF with diastolic function

In the total sample (n = 254), significant associations for all four diastolic parameters with the PF volume were found after adjusting for BMI, age, and sex. These data are depicted in Table 3. Analyses of the interactions with BMI, age, and sex, did not improve the model. In addition, in the extreme quartiles of PF volumes (n = 130) a significantly negative association between high PF and LAVI, high PF and e′ lateral, and high PF and TR, were found after adjusting for BMI, age, and sex. However, the difference in the mobility of the septal wall between the extreme quartiles of PF volume and between E/e′ the extreme quartile of PF volume were no longer evident after the model was adjusted for these factors. These regression data are depicted in Table 4.

**Table 3:** Multivariable linear regression analysis in the total population exploring associations between PF and parameters of diastolic cardiac function

	Unadjusted regression coefficient (95% CI)	p-value	Adjusted regression coefficient^a^ (95% CI)	p-value
Left atrial volume index (mL/m^2^)	− 0.24 (− 1.79; 1.32)	0.764	− 2.05 (− 3.92; − 0.19)	0.001
e′ septal (cm/s)	− 0.03 (− 0.52; 0.47)	0.917	− 0.13 (− 0.68; 0.43)	0.020
e′ lateral (cm/s)	− 0.21 (− 0.84; 0.41)	0.496	− 0.02 (− 0.71; 0.67)	< 0.001
E/e′	7.45 (6.49; 8.42)	0.335	0.16 (− 0.42; 0.74)	0.003
Tricuspid regurgitation (m/s)	0.04 (− 0.04; 0.12)	0.356	− 0.02 (− 0.12; 0.07)	0.001

*CI* confidence interval

^a^Adjusted for body mass index, age, and sex

**Table 4:** Multivariable linear regression analysis in the extreme PF quartiles (0 = low, 1 = high) exploring associations between PF and parameters of diastolic cardiac function

	Unadjusted regression coefficient (95% CI)	p-value	Adjusted regression coefficient^a^ (95% CI)	p-value
Left atrial volume index (mL/m^2^)	− 4.13 (− 7.47; − 0.80)	0.015	− 7.85 (− 12.13; − 3.56)	0.001
e′ septal (cm/s)	− 1.17 (− 2.25; − 0.10)	0.034	− 0.96 (− 2.28; 0.36)	0.088
e′ lateral (cm/s)	− 1.97 (− 3.33; − 0.60)	0.005	− 1.39 (− 3.13; 0.34)	0.020
E/e′	1.52 (0.40; 2.64)	0.009	1.33 (− 0.11; 2.77)	0.118
Tricuspid regurgitation (m/s)	0.06 (− 0.09; 0.22)	0.416	0.01 (− 0.18; 0.20)	0.004

*CI* confidence interval

^a^Adjusted for body mass index, age, and sex

### Distribution and determinants of the different components of the PF volume

**Fig. 5:**
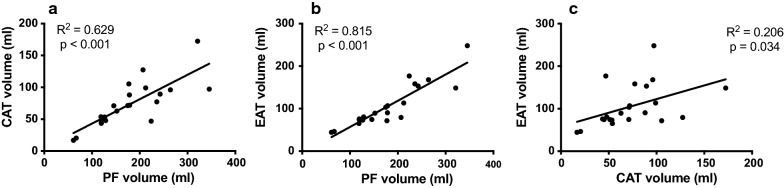
No relation of PF to its CAT and EAT component. The amount of CAT (**a**) and EAT (**b**) are not related to PF. Although EAT and CAT volume show a wide variation, they are linearly associated to each other (**c**), indicating that both increase with an increase of PF

In 10% of the total sample (n = 24), the EAT volume was studied by manually dividing the PF into the different CAT and EAT volumes. This random selection of 6 patients per PF quartile was made since the manual subdivision of the PF is extremely laborious, and to ascertain that the sample reflects the entire population. The data showed that with an increasing PF, no similar increase in the relative volume of EAT and CAT can be expected, as the relationship with the relative amount of EAT and CAT is lacking (p > 0.7). These data are illustrated in Fig. 5.

To gain further insight into whether EAT is the major culprit in hampering diastolic function as suggested because of its anatomic proximity to the myocardium, separate correlations of EAT were made with the different diastolic parameters. Despite the small number, a direct correlation of the percentage of EAT and e′ lateral was found. There was no correlation with EAT and the other diastolic function parameters (Additional file 1: Figure S4).

## Discussion

Studies associating PF or EAT with diastolic function are scarce and often contradictive. A partial explanation may be that most studies so far were performed in a non-healthy population, which may confound the reported associations of PF or EAT with diastolic function [15–20]. Here, we studied the association between PF and diastolic function in a lean to obese middle-aged population, with normal systolic and diastolic cardiac function. We evaluated these relationships independently of their metabolic profile as correction for metabolic risk factors was applied. Furthermore, we explored the association of EAT with PF and EAT with diastolic function.

We report that PF was significantly associated with the diastolic function parameters LAVI, e′ lateral, e′ septal, E/e′, and TR, when corrected for age, BMI, and sex. Adjustment for sex alone was already sufficient to render the association significant, since PF is different distributed between male and female. The reported associations indicate that even in our healthy population with a normal diastolic function, PF—independently of CVD risk factors related to age, BMI, and sex—is associated with diastolic function parameters.

In the analyses focusing on low and high PF volume, high PF was associated with a decrease in LAVI and e′ lateral, and an increase in TR (as depicted in Fig. 5). The decrease in e′ lateral is in line with previous research performed in (morbid) obese subjects with a high prevalence of T2DM [15]. The lower e′ lateral in the highest PF quartile reflects a slower relaxation of the lateral wall of the left ventricle, necessary for an effective diastolic filling phase. The lower LAVI in the highest PF quartile is not known to be a sign of lower diastolic function. We do not know what underlies these findings, but they may indicate that PF causes mechanical hindrance that compromises not only the mobility of the lateral left ventricle wall (e′ lateral), but also compresses the left atrium, and thereby reducing its volume (LAVI). This hypothesis needs further work.

Although EAT was only determined in a small subpopulation (n = 24), insights in the compartmental distribution of PF and its consequences on diastolic function can be gathered. We found that at increased PF volumes, the EAT and CAT compartments increased at a same amount relatively to the whole fat depot. This is surprising as Wu et al. reported that subjects after bariatric surgery showed a great loss of CAT and only a small decrease in EAT [37]. Therefore, the regional distribution of adipose tissue remains an important subject for further research, taking into account that this distribution plays an important role in the development of metabolic syndrome and CVD [38].

The association of high PF with e′ lateral suggests that in a healthy population the mechanical effects of PF limit the distensibility of the heart first, which subsequently contributes to diastolic dysfunction. This study suggests that secondly, after progression of this relaxation problem of the lateral wall, the LAVI might increase despite the compression of the PF mass, as seen in diastolic dysfunction. But this remains speculative, as we did not measure the mobility of the lateral wall of the left ventricle during the systolic phase. However, during systole the PF mass will be less restrictive than during diastole, which is in line with our hypothesis. Most notably, a mechanically limited heart is accompanied by pressure changes within the cardiomyocytes, which in turn can affect the metabolism of these cells, and thereby, negatively influence diastolic function.

Most of the research on PF so far focused on adipokine release and a potentially causal role in the formation of fibroses. Pressure changes due to increased PF leading to an altered metabolism are an alternative pathway how PF can influence cardiac function. Thus, although the underlying mechanism remains unknown, the idea that mechanical effects of high PF cause a diminished mobility of the myocardium is supported by the current data. As others already suggested, this diminished mobility may provoke fibrosis, which has been associated with diastolic dysfunction, however this remains to be elucidated. In our population changes in diastolic function parameters were associated with an increase in PF, however, the diastolic function was within normal range; hence no causality with fibrosis could be made.

### Limitations

As we performed a cross-sectional retrospective study, our study has some limitations by design. Due to the retrospective design, the low and high PF groups were not matched on all relevant characteristics. However, we did adjust our analyses for age, BMI, and sex. Although we corrected for age, BMI, and sex, some of metabolic characteristics such as glucose, HDL-cholesterol, and triglycerides, may confound the associations, although these metabolic characteristics were within normal range. Also, because of the retrospective design, there was timeframe of a maximum of three months between the CCTA and TTE, this may have influenced our association. In addition, since the manual subdivision of the PF is extremely laborious, we only separated the PF components in 10% of the total cohort, following random selection. Thus, the power was limited for exploring the metabolic effects of EAT, independently of PF, on diastolic function. The cross-sectional outline of this study does not allow any conclusions regarding possible causality. Future work should therefore include a prospective approach to evaluate causal relationships.

Finally, it is important to bear in mind that our study population consisted of relatively healthy subjects, whose cardiac diastolic function was considered to be good. We only studied the associations between PF and small variations in normal diastolic function, which also explains why we did not find correlations between the diastolic parameters and age, BMI, or sex, in our sample (Additional file 1: Figures S1, S2, S3). There were no subjects with clinically defined diastolic failure to assess the relationships between PF and diastolic dysfunction. This, of course, remains an important question for future research.

## Conclusion

The purpose of the current study was to determine the association of PF and cardiac diastolic function in a healthy population. Linear regression analysis revealed that PF, independently of age, BMI, and sex, is associated with the four diastolic ultrasound parameters which are decisive in the evaluation of diastolic function. A potential underlying mechanism of this may be that increased PF may compress the heart, leading to a limited distensibility in the diastole and fibrosis as seen in cardiac remodeling, and thus, may lead to diastolic dysfunction. This study adds to the growing body of research that explores possible mechanisms in the development of diastolic failure. Concluding, we confirm that PF, even in healthy subjects with normal cardiac function and without diabetes, does hinder diastolic function. The exact causality of this effect and the relationship with fibrosis remains to be determined.

## Supplementary information

**Additional file 1.** Supplementary figures.

## Data Availability

The datasets used and/or analysed during the current study are available from the corresponding author on reasonable request.

## Abbreviations

ASE: American Society of Echocardiography
BMI: Body Mass Index
CABG: Coronary Artery Bypass Grafting
CAT: Cardiac Adipose Tissue
CCTA: Computed Tomographic Angiography
CI: confidence interval
CVD: cardiovascular disease
e′: lateral mobility of the lateral ventricle wall
e′: septal mobility of the septal ventricle wall
EAT: Epicardial Adipose Tissue
EACVI: European Association of Cardiovascular Imaging
IQR: interquartile range
LAVI: Left Atrial Volume Index
LV: left ventricle
LVEF: Left Ventricular Ejection Fraction
PF: pericardial fat
T2DM: type 2 diabetes mellitus
TR: peak velocity of tricuspid regurgitation
