# Correction to: Pericardial fat and its influence on cardiac diastolic function

**DOI:** 10.1186/s12933-024-02346-4

**Published:** 2024-07-24

**Authors:** Vera H. W. de Wit‑Verheggen, Sibel Altintas, Romy J. M. Spee, Casper Mihl, Sander M. J. van Kuijk, Joachim E. Wildberger, Vera B. Schrauwen‑Hinderling, Bas L. J. H. Kietselaer, Tineke van de Weijer

**Affiliations:** 1 NUTRIM School of Nutrition and Translational Research in Metabolism, Maastricht University, Maastricht, Netherlands; 2 Department of Nutrition and Movement Sciences, Maastricht University Medical Center, Maastricht, Netherlands; 3 Department of Cardiology, Maastricht University Medical Center, Maastricht, Netherlands; 4 Department of Radiology and Nuclear Medicine, Maastricht University Medical Center, Maastricht, Netherlands; 5 CARIM School for Cardiovascular Diseases, Maastricht University Medical Center, Maastricht, Netherlands; 6 Department of Clinical Epidemiology and Medical Technology Assessment, Maastricht University Medical Center, Maastricht, Netherlands

**Correction to: Cardiovascular Diabetology (2020) 19:129** 10.1186/s12933-020-01097-2

Following publication of the original article [1], the authors have inadvertently made a few minor mistakes in the figure and tables.

First, Fig. 5 is incorrect, as the absolute instead of the relative volumes are depicted. The legend refers to the figure below, indeed showing no relationship between the absolute increase of PF and the relative (%) increase of CAT and EAT.

**Correct version of Fig. 5:**
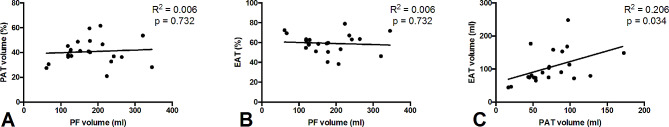
Showing no relationship of increasing volume of PF to the relative contribution (%) of the CAT and EAT component (**a**) and (**b**), hence the contribution of both compartments remains equal despite different volumes of total PF. EAT and CAT volume show a wide variation, they are linearly associated to each other (**c**), indicating that both increase with an increase of PF.

The figure in the paper shows the absolute volumes, which therefore are linearly correlated with the total PF volume. The capitation and text below the figure however, refer to the new figure above.

**Old figure**:
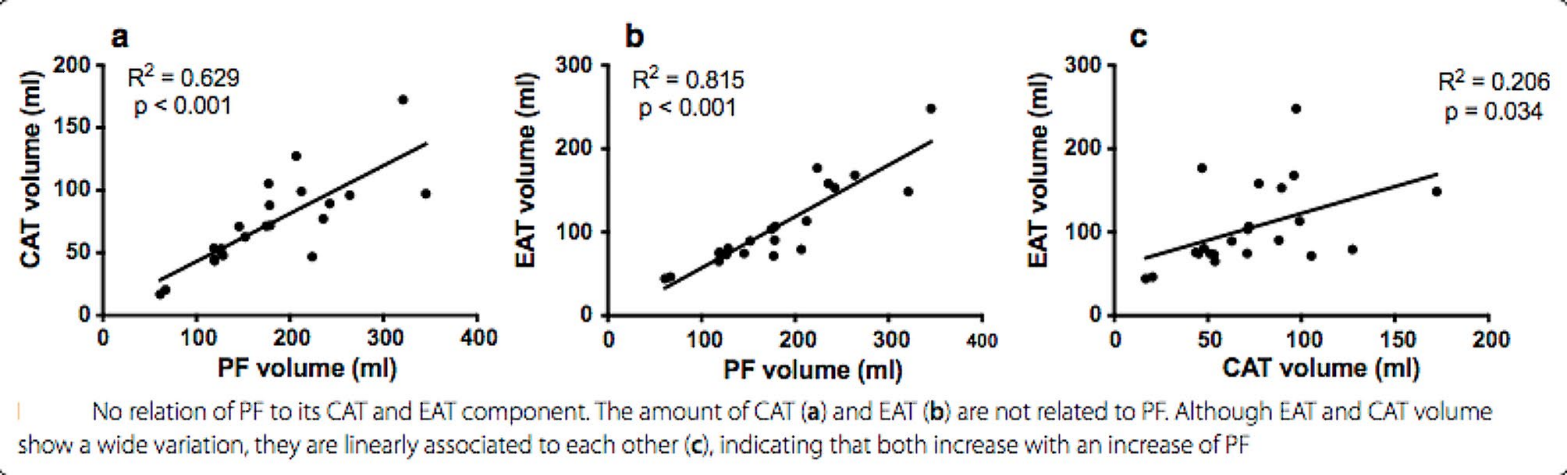


The second mistake involves the p-values of Tables 3 and 4. Although the data and the 95% CI intervals are correct, and hence significance could have been concluded from the data, unfortunately the reported p-values were incorrect. Below we have corrected the tables and added the correct P-values to the tables. After correction of the p-values, only the Left atrial volume index (LAVI) is associated with the PF after correction for age, BMI and gender and the correlation of PF with the other parameters of diastolic function is lost. As a result, part of the results may be biased by age, gender and BMI. Nonetheless, the mechanical effect on the left atrium remains and the changed results do not alter the discussion and/or the message of the paper.

**Table 3:** Multivariable linear regression analysis in the total population exploring associations between PF and parameters of diastolic cardiac function

		*p*-value	Adjusted regression coefficient^a^ (95% CI)	Original *p*-value	Correct *p*-value
Left atrial volume index (mL/m^2^)	− 0.24 (− 1.79; 1.32)	0.764	− 2.05 (− 3.92; − 0.19)	0.001	0.031
e′ septal (cm/s)	− 0.03 (− 0.52; 0.47)	0.917	− 0.13 (− 0.68; 0.43)	0.02	0.646
e′ lateral (cm/s)	− 0.21 (− 0.84; 0.41)	0.496	− 0.02 (− 0.71; 0.67)	< 0.001	0.956
E/e′	7.45 (6.49; 8.42)	0.335	0.16 (− 0.42; 0.74)	0.003	0.433
Tricuspid regurgitation (m/s)	0.04 (− 0.04; 0.12)	0.356	− 0.02 (− 0.12; 0.07)	0.001	0.631

**Table 4:** Multivariable linear regression analysis in the extreme PF quartiles (0 = low, 1 = high) exploring associations between PF and parameters of diastolic cardiac function

	Unadjusted regression coefficient (95% CI)	*p*-value	Adjusted regression coefficient^a^ (95% CI)	Original *p*-value	Correct *p*-value
Left atrial volume index (mL/m^2^)	− 4.13 (− 7.47; − 0.80)	0.015	− 7.85 (− 12.13; − 3.56)	0.001	< 0.001
e′ septal (cm/s)	− 1.17 (− 2.25; − 0.10)	0.034	− 0.96 (− 2.28; 0.36)	0.088	0.149
e′ lateral (cm/s)	− 1.97 (− 3.33; − 0.60)	0.005	− 1.39 (− 3.13; 0.34)	0.02	0.113
E/e′	1.52 (0.40; 2.64)	0.009	1.33 (− 0.11; 2.77)	0.118	0.472
Tricuspid regurgitation (m/s)	0.06 (− 0.09; 0.22)	0.416	0.01 (− 0.18; 0.20)	0.004	0.910

These two errors had no effect on the scientific content or conclusions of the paper. We deeply apologize that this occurred, and take responsibility for the error and hereby present the correction.
