# Supplementary material for: Pericardial fat and its influence on cardiac diastolic function
Source: Cardiovasc Diabetol. 2020 Aug 17;19:129. doi: 10.1186/s12933-020-01097-2 (PMC7430122; doi:10.1186/s12933-020-01097-2)
Supplement: Supplementary file 1 — Additional file 1. Supplementary figures. [file 12933_2020_1097_MOESM1_ESM.docx]

***Supplementary***


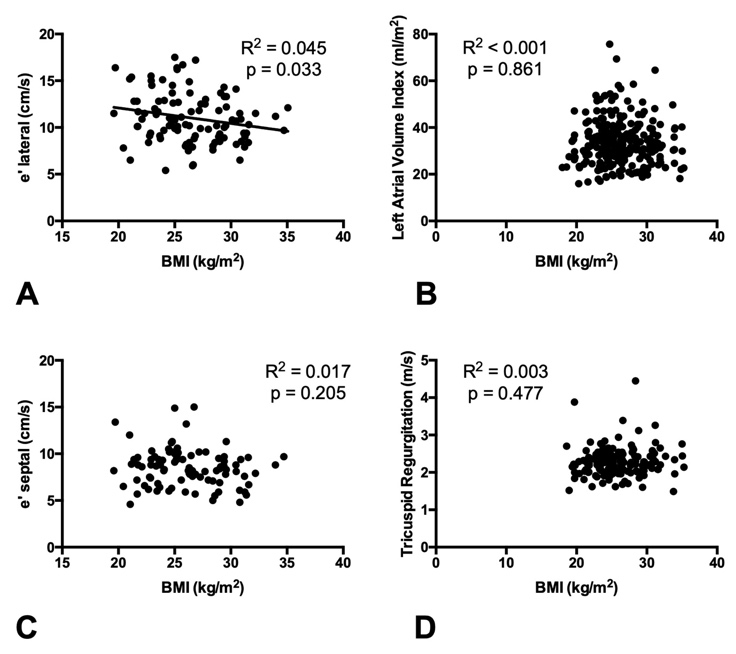


**Fig. S1. Associations between BMI and diastolic function parameters in a healthy population.** BMI is negatively associated with e’ lateral (A), but not with LAVI (B), e’ septal (C) or TR (D) in a healthy population.


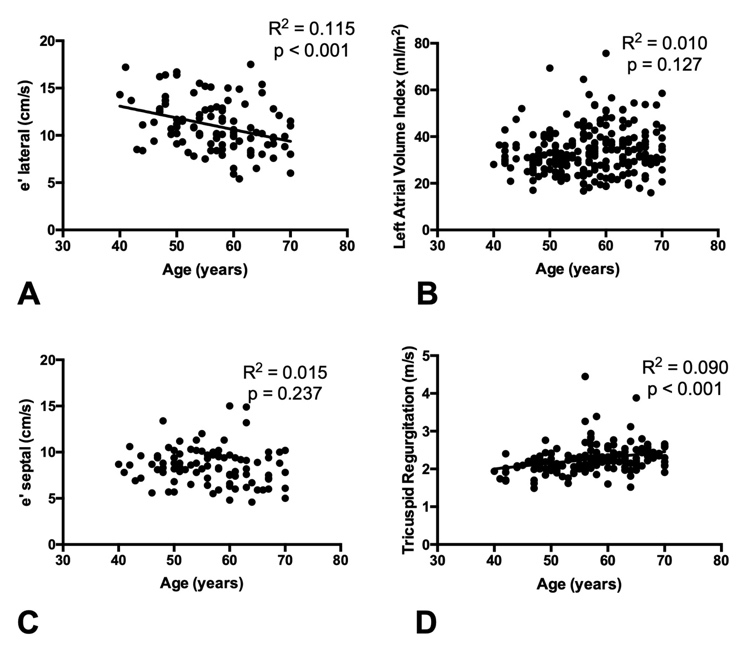


**Fig. S2. Associations between age and diastolic function parameters in a healthy population.** Age is negatively associated with e’ lateral (A), and positively associated with TR (D) in healthy population. Age is not with LAVI (B), or e’ septal (C).


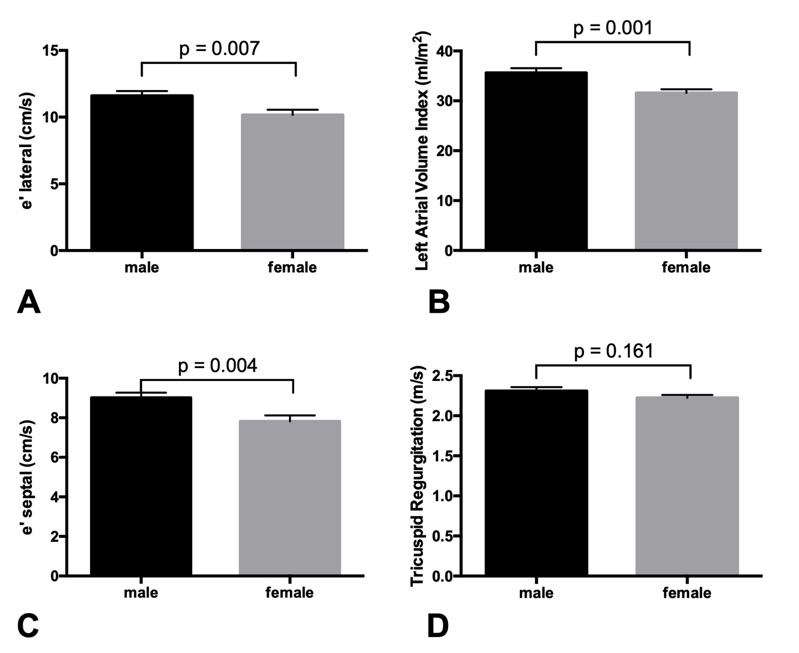


**Fig. S3. Associations between sex and diastolic function parameters in a healthy population.** Males are associated with higher e’ lateral (A), higher LAVI (B), and higher e’ septal (C), compared to females. No sex difference is observed in TR (D).


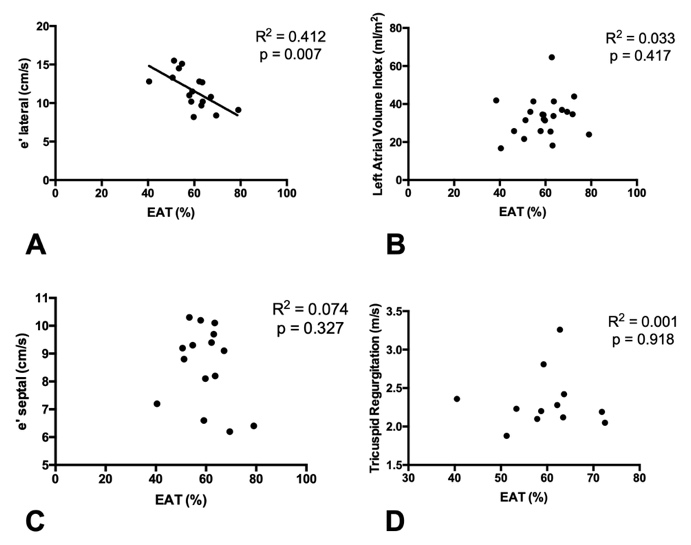


**Fig. S4. Associations between EAT volume (as % of PF volume) and diastolic function parameters in subjects with low or high PF volume within a healthy population.** Only e’ lateral (B) was associated with the relative amount of EAT volume; no associations were found for LAVI (B), e’ septal (C), nor TR (D).
